# Advances in Vaccine Development of the Emerging Novel Genotype Fowl Adenovirus 4

**DOI:** 10.3389/fimmu.2022.916290

**Published:** 2022-05-20

**Authors:** Aijing Liu, Yu Zhang, Hongyu Cui, Xiaomei Wang, Yulong Gao, Qing Pan

**Affiliations:** ^1^State Key Laboratory of Veterinary Biotechnology, Harbin Veterinary Research Institute of Chinese Academy of Agricultural Sciences, Harbin, China; ^2^Jiangsu Co-Innovation Center for Prevention and Control of Important Animal Infectious Disease and Zoonoses, Yangzhou University, Yangzhou, China

**Keywords:** HHS, vaccine advances, emerging, FAdV-4, novel genotype

## Abstract

Fowl adenovirus (FAdV) was first reported in Angara Goth, Pakistan, in 1987. For this reason, it is also known as “Angara disease.” It was later reported in China, Japan, South Korea, India, the United States, Canada, and other countries and regions, causing huge economic losses in the poultry industry worldwide. Notably, since June 2015, a natural outbreak of severe hydropericardium hepatitis syndrome (HHS), associated with a hypervirulent novel genotype FAdV-4 infection, has emerged in most provinces of China. The novel virus FAdV-4 spread rapidly and induced a 30-100% mortality rate, causing huge economic losses and threatening the green and healthy poultry breeding industry. Vaccines against FAdV-4, especially the emerging novel genotype, play a critical role and will be the most efficient tool for preventing and controlling HHS. Various types of FAdV-4 vaccines have been developed and evaluated, such as inactivated, live-attenuated, subunit, and combined vaccines. They have made great contributions to the control of HHS, but the details of cross-protection within FAdVs and the immunogenicity of different vaccines require further investigation. This review highlights the recent advances in developing the FAdV-4 vaccine and promising new vaccines for future research.

## Introduction

Fowl adenovirus (FAdV) belongs to the family Adenoviridea and genus *Aviadenovirus* and is further divided into five species (FAdV-A-E) with 12 serotypes (FAdV-1-8a, 8b-11) based on the profile of restriction enzyme digestion and sera cross-neutralization assay according to the guidelines of the International Committee on Taxonomy of Viruses (1). FAdVs are capable of infecting various birds, such as chickens (2, 3), ducks (4–6), geese (7), pigeons (8), peacocks (9), and other wild birds, inducing severe clinical symptoms or potential infection. FAdVs show different tropisms for multiple organs and are associated with several high-impact poultry diseases, such as hydropericardium-hepatitis syndrome (HHS), inclusion body hepatitis (IBH), and gizzard erosion (GE). FAdV infection associated with HHS, IBH, and GE has been reported worldwide and induces huge economic losses in the poultry industry.

HHS associated with serotype 4 fowl adenovirus (FAdV-4) infection was first found and reported in Angara Goth, a region of Pakistan, in 1987, so the disease is also known as “Angara disease” (10). It was later reported in China (3, 11), Japan (12, 13), South Korea (14), India (15), the United States (16), Canada (17), and other countries and regions, causing huge economic losses in the poultry industry worldwide. Notably, since June 2015, a natural outbreak of severe HHS associated with a hypervirulent novel genotype FAdV-4 infection has emerged in most provinces of China (3, 11). The novel genotype FAdV-4 spread rapidly and induced a 30-100% mortality rate, causing huge economic losses and threatening the green and healthy poultry breeding industry. Furthermore, co-infections of FAdV-4 with other virus further aggravate the harmfulness of the disease (18–20).

Currently, various types of vaccines have been developed and evaluated to control HHS, and several inactivated combined vaccines have been licensed and produced by commercial companies, which have made important contributions to the poultry industry. Given the ability to deliver foreign adenovirus genes, natural non-pathogenic FAdV-4 (16) and artificial attenuated FAdV-4 (17) have developed efficient vaccine vectors, which protect chickens against HHS and significantly reduce the cost of the combined vaccine. This brief review summarizes vaccine development efforts using inactivated viruses, live-attenuated viruses, subunit antigens, and combined vaccines against FAdV-4, especially novel FAdV-4.

### Inactivated Monovalent Vaccines

Traditional inactivated vaccine immunization remains the main preventive method for some poultry diseases, especially emerging viruses such as SARS-CoV-2 (21, 22). Given the emergence of the hypervirulent novel genotype FAdV-4, inactivated vaccine immunization remains the main prevention method, having the advantages of safety, cost-saving, good humoral immunity effect, and low influence by maternal antibodies. For the emerging HHS, Pan et al. isolated and identified the HLJFAd15 strain as a novel hypervirulent FAdV-4 from the field layers with HHS in China in 2015 (2). Subsequently, SPF chickens were immunized with an inactivated oil-emulsion FAdV-4 vaccine formulated with the HLJFAd15 strain, and the vaccine’s protective effect was evaluated (23). The results indicated that the vaccine could provide a high-level antibody, preferential T helper 2 (Th2) response, and full protection against a lethal dose of novel hypervirulent FAdV-4. Similarly, the novel genotype SDJN0105 strain (24), HN strain (25), and CH/GZXF/1602 strain (26) developed an inactivated vaccine, and all vaccines induced high levels of antibodies and showed sufficient protection for chickens from HHS.

Du et al. attempted to develop vaccines from both embryo-adapted and cell culture-derived viruses to optimize the manufacturing technique of inactivated vaccines (25). The results showed no mortality in either of the immunized groups in the challenge experiment. However, cell-culture-derived vaccines could induce earlier and higher humoral immune responses and no lesions appeared, but IBH was observed in the embryo-adapted derived vaccine, indicating that cell-culture-derived vaccines could be a better candidate to control emerging HHS.

For cross-protection of the FAdV-4 inactivated vaccine, Kim et al. developed an inactivated vaccine with a traditional FAdV-4 strain K531/07 and evaluated the cross-protection of the vaccine against various serotypes of FAdV causing IBH or HHS, including serotypes 5, 8a, 8b and 11 (27). They found that the inactivated FAdV-4 vaccine could provide cross-protection against various serotypes of FAdV in vaccinated chickens and progenies of vaccinated breeders, indicating that the FAdV-4 vaccine could be an effective candidate for the prevention of IBH and HHS. Furthermore, Xia et al. prepared an inactivated vaccine using a novel genotype FAdV-4 strain CH/GZXF/1602, and the immunogenicity evaluation results showed that the FAdV-4 vaccine could protect chickens against both virulent FAdV-4 and virulent FAdV-8b (26).

Notably, all inactivated vaccines were derived from wild-type virulent FAdV-4 strains. As biosafety threats of inactivated vaccines from potential pathogenic components have been presented to the poultry industry, safer vaccines are urgently needed. Zhang et al. replaced the virulent vaccine formulation with an artificial non-pathogenic FAdV-4 strain rHN20 using a reverse genetic technique and developed a novel inactivated oil-adjuvanted vaccine derived from the rHN20 strain (28). The results showed that the vaccine-induced high titers of neutralizing antibodies provided full protection from a lethal dose of virulent FAdV-4 challenge and significantly reduced potential biosafety threats. The details of inactivated vaccines are summarized in **Table 1**.

**Table 1:** List of inactivated and live-attenuated vaccines against FAdV-4.

Inactivated vaccine						Live-attenuated vaccine				
Strain	Origin	Adjuvant	Dosage (/bird)	Immune route	Survival rate (%)	Strain	Origin	Dosage (/bird)	Immune route	Survival rate (%)
HLJFAd15	CEL^a^	Oil	10^6^ TCID_50_	IM^f^	100	FAdV-4/QT35	QT35	5×10^4^ TCID_50_	IM	100
CH/GZXF/1602	CEK^b^	Oil	4.5×10^5^ TCID_50_	H^g^	100	rR188I	LMH	10^5^ PFU	IM	100
SDJN0105	CEF^c^	Oil	10^6^ TCID_50_	IM	100	rHN20	LMH	10^6^ PFU	IN	100
HN	LMH^d^	Oil	10^6^ TCID_50_	H^h^	100	rHN20	LMH	10^6^ PFU	IM	100
HN	CE^e^	Oil	10^6^ TCID_50_	H	100 (IBH)	rHN20	LMH	10^6^ PFU	H	100
K531/07	CEL	Seppic ISA 70	5×10^5^ TCID_50_	IM	80	FAdV4-RFP_F1	LMH	2×10^5^ TCID_50_	IM	100
rHN20	LMH	Oil	3×10^6^ PFU	IM	100	FA4-EGFP	LMH	10^6^ TCID_50_	IM	100
rHN20	LMH	Oil	3×10^6^ PFU	H	100	FAdV4-EGFP-rF2	LMH	2.5×10^4^ TCID_50_	IM	100

a Chicken embryo liver cells.

b Chicken embryo kidney cells.

c Chicken embryo fibroblast cells.

d Leghorn male hepatocellular cells.

e Chicken embryo.

f Intramuscular.

g Subcutaneous.

h Intranasal.

### Live-Attenuated Monovalent Vaccines

For the traditional genotype FAdV-4, Schonewille et al. developed an attenuated FAdV-4 vaccine by adapting a pathogenic virus to the QT35 cell line, and no clinical signs or mortality were observed in birds challenged with the attenuated virus (29). Although enzyme-linked immunosorbent assay (ELISA) and neutralization tests indicated a weak antibody response in some birds following immunization with the live vaccine, the vaccine provided full protection against the challenge, which is an interesting phenomenon for FAdVs.

For the novel genotype FAdV-4, Zhang et al. identified the critical gene and amino acid (aa) for virus virulence (30), subsequently, two non-pathogenic strains, rHN20 and rR188I, were rescued by the reverse genetic technique based on the fosmid system. rHN20 caused no clinical signs or mortality, indicating that hexon determines the virulence of FAdV-4. Furthermore, Zhang et al. identified aa 188R of hexon as the critical aa for virulence of the novel genotype FAdV-4, and the rR188I strain also showed no pathogenicity in SPF chickens. The immunogenicity of both rHN20 (28) and rR188I (30) was evaluated in SPF chickens when they were used as live-attenuated vaccines. Chickens inoculated with different doses and routes of rHN20 or rR188I produced high levels of neutralizing antibodies and were fully protected against a lethal dose challenge of the pathogenic novel genotype FAdV-4. Furthermore, the immunized groups showed no clinical symptoms or histopathological changes, and the viral load was significantly lower after the challenge.

Not only was the hexon-edited virus significantly attenuated, but the fiber-1 and fiber-2 edited viruses were rescued and identified as non-pathogenic to SPF chickens. Mu et al. rescued the recombinant virus FAdV4-RFP_F1 *via* the CRISPR/Cas9 technique, which contained a fusion protein of RFP and fiber-1 (31). The recombinant virus was efficiently attenuated and provided full protection against the lethal challenge of FAdV-4, demonstrating that fiber-1-edited FAdV4-RFP_F1 could be a live-attenuated vaccine candidate and fiber-1 could be a potential insertion site for novel vaccine development. In addition, Xie et al. generated a recombinant virus FA4-EGFP expressing EGFP-fiber-2 fusion protein *via* the CRISPR/Cas9 technique (32). FA4-EGFP caused no clinical signs or mortality in chickens, indicating that the virus was significantly attenuated.

Moreover, FA4-EGFP could also provide efficient protection against a lethal dose challenge, suggesting that fiber-2 edited FA4-EGFP is a vaccine candidate and a potential insertion site for delivering foreign antigens. Furthermore, Xie et al. replaced the *fiber-2* gene with *egfp* and rescued FAdV-4-EGFP-rF2, which could efficiently replicate in LMH-F2 cell lines expressing fiber-2 protein (33). FAdV-4-EGFP-rF2 was highly attenuated and provided full protection against the pathogenic FAdV-4 challenge. The FAdV-4-EGFP-rF2 vaccine-induced neutralizing antibodies are at the same level as FA4-EGFP. The details of live-attenuated monovalent vaccines are summarized in **Table 1**.

### Subunit Monovalent Vaccines

Subunit vaccines are safer than vaccines based on the whole virus in production and vaccination procedures, and researchers have made important efforts to develop subunit vaccines against FAdV-4. Different subunit antigens, expression systems, and adjuvants have been evaluated to develop FAdV-4 subunit vaccines.

Before the novel genotype FAdV-4, several studies tried to develop a subunit vaccine of the traditional genotype FAdV-4. Shah et al. utilized a prokaryotic expressed structure protein penton base coupled with Freund’s complete adjuvant (FCA) to develop a subunit vaccine that showed 90% protection for chickens (34). Aziz et al. evaluated both the prokaryotic expressed full-length and epitope-focused 1-225 aa of penton formulated with Montanide ISA 71VG adjuvant, and both vaccines showed protection rates of 50% (35). Schachner et al. compared the immunogenicity of different capsid proteins of FAdV-4, including fiber-1, fiber-2, and L1 region of hexon (L1-hexon) (36). Fiber-1, fiber-2, and L1-hexon were simultaneously expressed in the baculovirus system and composed of GERBU Adjuvant LQ no.3000, and fiber-2 (27/28) showed better protection efficacy than fiber-1 (16/26) and L1-hexon (7/26), highlighting that fiber-2 might be an ideal antigen component for subunit vaccine development. Shah et al. first tried a prokaryotic-expressed non-structural protein 100 K of FAdV-4 coupled with FCA; unfortunately, it showed only a 40% protection rate (37).

Although HHS associated with a novel genotype FAdV-4 emerged in 2015, great progress has been made in subunit vaccine development. Wang et al. tested the efficacy of FAdV-4 surface protein fiber-1, fiber-2, L1-hexon, and penton base expressed in *Escherichia coli* and formulated with FCA (38). The results indicated that fiber-2 (50 µg/bird) and fiber-1 (100 µg/bird) could induce complete protection, while protection of the L1-hexon and penton bases could induce considerable protection at high dosages but not completely. The good immunogenicity of prokaryotic expressed fiber-2 was further confirmed when fiber-2 was coupled with different adjuvants, such as FCA (39), Montanide ISA 71VG (40), and Sigma adjuvant (41), respectively. However, fiber-1 showed better protection than fiber-2 when expressed in a eukaryotic system, although the protection rates of both fiber-1 and fiber-2 were higher than those of the penton base (42). Wang et al. utilized a commercial adenovirus vector to express antigens in HEK293 cells, and the antigen was assembled into a penton-dodecahedron (Pt-Dd), which provided 100% protection (42). Jia et al. constructed a recombinant *Lactococcus lactis* and *Enterococcus faecalis* expressing truncated hexon protein or fused with a dendritic cell-targeting peptide, which was orally immunized and induced protection rates varying from 50 to 90% (43). These studies provide a solid theoretical basis for the research and development of subunit vaccines.

In addition, several studies have been conducted to obtain many results. Yin et al. constructed a pVAX1-Fiber2 DNA vaccine, but the protection was also insufficient (60%) for the novel genotype FAdV-4, and the above studies require further optimization (41). Nevertheless, many improvements have achieved good results. Hu et al. developed a fusion subunit antigen of fiber-2 (Gly275- Pro479) and hexon (Met21-Val51) that was capable of providing full protection for chickens (44). Tufail et al. displayed several highly conserved epitopic regions of hexon on the virus-like particle (VLP) of the core protein of the hepatitis B virus (45). The VLP vaccine containing Asp348-Phe369, Ser19-Pro82, and Gly932-Phe956 confer 90%, 70%, and 40% protection. The details of subunit vaccines against FAdV-4 are summarized in **Table 2**.

**Table 2:** List of subunit vaccines against FAdV-4.

Subunit vaccine						
Antigen	Strain	Expression system	Dosage (/bird)	Adjuvant	Immune route	Survival rate (%)
penton	PR-06	*E. coli* ^e^	25 μg	FCA^h^	H ^i^	90
penton	NIAB/NIBGE 01	*E. coli*	100 μg	Montanide ISA 71VG	H	50
penton (1-225 aa^a^)	NIAB/NIBGE 01	*E. coli*	100 μg	Montanide ISA 71VG	H	50
fiber-1	KR5	Baculovirus	50 μg	GERBU Adjuvant LQ no.3000	IM ^j^	61.5
fiber-2	KR5	Baculovirus	50 μg	GERBU Adjuvant LQ no.3000	IM	96.4
L1-hexon^b^	KR5	Baculovirus	50 μg	GERBU Adjuvant LQ no.3000	IM	26.9
100K	NIAB/NIBGE 01	*E. coli*	25 μg	FCA	H	40
fiber-1	SXD15	*E. coli*	100 μg 50 ug	FCA FCA	IM IM	100 75
fiber-2	SXD15	*E. coli*	50 μg	FCA	IM	100
L1-hexon	SXD15	*E. coli*	50 μg	FCA	IM	70
penton	SXD15	*E. coli*	50 μg	FCA	IM	35
fiber-2	HB1501	*E. coli*	2.5 μg	FCA	H	100
fiber-2	–	*E. coli*	10 μg	Montanide ISA 71VG	IM	100
fiber-2	GZ-QL	*E. coli*	150 μg 100 ug 50 ug	Sigma Sigma Sigma	IM IM IM	100 100 80
fiber-1	SXD15	Ad-HK2	0.5 μg	N/A^l^	IM	100
fiber-2	SXD15	Ad-HK2	0.5 μg	N/A	IM	80
penton	SXD15	Ad-HK2	0.5 μg	N/A	IM	67.7
PtDd^c^	SXD15	Ad-HK2	0.5 μg	N/A	IM	100
DC-hexon^d^	GX01	*L. lactis* ^f^	10^10^ CFU	N/A	O ^k^	60
hexon	GX01	*L. lactis*	10^10^ CFU	N/A	O	50
DC-Hexon	GX01	*E. faecalis* ^g^	5×10^9^ CFU	N/A	O	90
hexon	GX01	*E. faecalis*	5×10^9^ CFU	N/A	O	80

a Amino acid.

b Loop 1 region of hexon.

c Penton-dodecahedron.

d DC-Hexon: hexon fused with dendritic cell targeting peptide.

e Escherichia coli.

f Lactococcus lactis.

g Enterococcus faecalis.

h Freund’s complete adjuvant.

i Subcutaneous.

j Intramuscular.

k Oral.

l Not applicable.

### Combined Vaccines

Adenovirus, especially the commercial human adenovirus, is an efficient and stable delivery carrier widely used for gene therapy (46) and vaccines against emergent viruses, such as Ebola (47) and SARS-CoV-2 (48). With gene editing and reverse genetic technology, the emerging novel genotype, FAdV-4, has been successfully modified as a viral vector and applied to construct combined vaccines. Pan et al. firstly developed a novel genotype, FAdV-4, and identified the natural large genomic 1966bp deletion as a foreign gene insertion site (49). Mu et al. also optimized the CRISPR/Cas9 operating platform to insert a foreign gene into the fiber gene of FAdV-4 (31). Meanwhile, Yan et al. established an easy-to-use reverse genetics system based on Gibson assembly to modify the right and partial left genes (50). Pei et al. developed an infectious clone based on cosmid and found that ORF16 and 17 were important for virus replication and unsuitable for foreign gene insertion (51). Finally, Pan et al. built a reverse genetic technique based on the fosmid system to operate the genome of the novel genotype FAdV-4 and further identified 10 ORFs at the left end and 13 ORFs at the right end of the novel FAdV-4 as non-essential regions for virus replication, which provided a good foundation for foreign gene delivery (52).

Virulence weakening is critical for vector development because of the high pathogenicity of the novel genotype FAdV-4. Zhang et al. first identified the critical gene and key aa for the virulence of the novel FAdV-4 (30) and subsequently obtained the non-pathogenic strain rHN20, posting the basis for development and application. Subsequently, the immunogenetic VP2 gene of the very virulent infectious bursal disease virus (vvIBDV) was inserted into the natural 1966Del deletion site and induced complete protection in chickens against both the novel genotype FAdV-4 and vvIBDV challenge when used as an inactivated vaccine (53) or live vectored vaccine (52). Lu et al. inserted a FAdV-8b fiber into the fiber-2 position of FAdV-4, which simultaneously protected chickens from novel genotype FAdV-4 induced HHS and FAdV-8b induced IBH (54).

In addition to the above FAdV-4 vectored vaccines, several other combined vaccines were evaluated and showed good effects. Luca et al. constructed a chimeric fiber protein (crecFib-4/11) capable of simultaneously protecting chickens against HHS and IBH (55), highlighting a new concept: chimeric fiber vaccines can be extended across viral species. Tian et al. developed a recombinant Newcastle disease virus (NDV) LaSota vaccine strain expressing fiber-2 of FAdV-4 (rLaSota-fiber2) and live and inactivated vaccines derived from rLaSota-fiber2 (56). Both vaccines provided complete protection against virulent NDV. However, the live rLaSota-fiber2 vaccine provided better protection against the FAdV-4 challenge than the inactivated vaccine, indicating that the NDV-vectored FAdV-4 vaccine is a promising bivalent vaccine candidate to control both HHS and ND.

## Conclusions

Although various vaccines against the novel genotype FAdV-4 have been developed, and great progress has been made in controlling emerging HHS, many studies need further investigation. Preliminary evaluation of the inactivated vaccine derived from the traditional genotype FAdV-4 has been conducted in cross-protection with other serotypes of FAdV, but cross-protection vaccines based on the novel genotype FAdV-4 need systemic analysis within different serotypes of FAdV. Furthermore, the FAdV-4 vaccine vector needs to be deeply explored as an efficient vector to deliver multiple foreign antigens to reduce vaccine costs further. Meanwhile, studies on novel vaccine adjuvants, subunit antigens, and new vaccines such as DNA or mRNA vaccines against the novel genotype FAdV-4 have been limited, and more research is needed on the control of HHS.

## Author Contributions

QP and YG conceived of and designed the study. AL and YZ conducted the literature search, analysis, and draft-writing. HC and XW helped write the research questions in the first draft and the modifications. All the authors contributed to the study and approved the final manuscript for publication.

## Funding

This study was supported in part by the National Natural Science Foundation of China (32072879), the China Agriculture Research System (CARS-41-G15), and the Natural Science Foundation of Heilongjiang Province (TD2019C003).

## Conflict of Interest

The authors declare that the research was conducted without potential commercial or financial relationships construed as a potential conflict of interest.

## Publisher’s Note

All claims expressed in this article are solely those of the authors and do not necessarily represent those of their affiliated organizations, or those of the publisher, the editors and the reviewers. Any product that may be evaluated in this article, or claim that may be made by its manufacturer, is not guaranteed or endorsed by the publisher.
